# Supplementary material for: Cat–Owner Relationship and Cat Behaviour: Effects of the COVID-19 Confinement and Implications for Feline Management
Source: Vet Sci. 2022 Jul 18;9(7):369. doi: 10.3390/vetsci9070369 (PMC9324668; doi:10.3390/vetsci9070369)
Supplement: Supplementary file 1 [file vetsci-09-00369-s001.zip › vetsci-1790879-supplementary.pdf]

Annex S1: complete questionnaire translated from Italian into English
Pets during the COVID confinement

---

1) If you currently live in Italy and agree to participate in this study, please mark the "Yes" option.*
( ) Yes

( ) No

General information

2) What age group do you belong to?*
( ) Under 18 yoa

( ) 18-25 yoa

( ) 26-35 yoa

( ) 36-45 yoa

( ) 46-55 yoa

( ) 56-65 yoa

( ) 66-75 yoa

( ) 76+ yoa

3) What is your gender?*
( ) Male

( ) Female

( ) Other

4) Education level?

( ) Middle

( ) High

( ) Graduate

( ) Post-graduate

( ) Other

5) What is your employment?

( ) I work with animals (Veterinarian, dog trainer, etc…)

( ) Freelancer

( ) Student

( ) Labourer

( ) Retired

( ) Stay-at-home

( ) Other

6) How many people live in the household at this time? (If there is none in one or more categories, don't forget to select "none").*

	**None**	**1**	**2**	**3**	**4**	**5**	**6 or more**
Number of children aged 5 years old or under?	( )	( )	( )	( )	( )	( )	( )
Number of children aged 6 to 12 years old?	( )	( )	( )	( )	( )	( )	( )
Number of children aged 13 to 17 years old?	( )	( )	( )	( )	( )	( )	( )
Number of adults aged 18 to 64 years old?	( )	( )	( )	( )	( )	( )	( )
Number of adults aged 65 years or older?	( )	( )	( )	( )	( )	( )	( )

7) What is your role in the household? (Please choose the option that most applies)*

( ) Child

( ) Parent

( ) Grandparent

( ) Adult without children

( ) Other

8) How many dogs and/or cats live in your household?*

	**None**	**1**	**2**	**3**	**4**	**5**	**6 or more**
Dogs	( )	( )	( )	( )	( )	( )	( )
Cats	( )	( )	( )	( )	( )	( )	( )

9) What type of home do you live in?*
( ) A house
( ) A flat or apartment

10) Does your home have any outdoor space (please tick all that apply)*
( ) No

( ) Yes, it has a common outdoor place
( ) Yes, it has an outdoor terrace
( ) Yes, it has a garden/private yard
( ) Yes, it has a balcony

11) Please indicate the zip code of the place where you live

____

12) How big is your home?*
( ) Large
( ) Medium sized
( ) Small

13) How do you rate your family's total income?
( ) Low
( ) Medium
( ) High

14) The official confinement applies differently to people. For example, some people are authorised to leave home to work. Please select the option that best describes your household situation

( ) We are all confined and none of us work from home.
( ) We are all confined and some of us work from home
( ) We are all confined and we all work from home
( ) Some of us are confined, and some of us have permission to go out to work
( ) We all have permission to go out to work

15) How many weeks have you been confined at home since the official announcement of movement restrictions?*
( ) 1 week
( ) 2 weeks
( ) 3 weeks
( ) 4 weeks
( ) 5 weeks
( ) 6 weeks
( ) 7 weeks
( ) 8 weeks
( ) 9 weeks
( ) 10 weeks
( ) 11 weeks

16) What negative impact has the official confinement had on your household?*

	A lot	Quite a lot	Somewhat	Little	Very little	None
Financial impact	( )	( )	( )	( )	( )	( )
Emotional impact	( )	( )	( )	( )	( )	( )

Health impact	( )	( )	( )	( )	( )	( )
Lifestyle impact	( )	( )	( )	( )	( )	( )

17) To what extent has confinement affected your quality of life?*
( ) It is much better
( ) It is moderately better
( ) It is slightly better
( ) It is no different
( ) It is slightly worse
( ) It is moderately worse
( ) It is much worse

Questions about one of your pets

If you own more than one cat, please answer for the one whose name is first in alphabetical order.

18) What is the age of your cat in years?
( ) less than 1 year
( ) 1 year
( ) 2 years
( ) 3 years
( ) 4 years
( ) 5 years
( ) 6 years
( ) 7 years
( ) 8 years
( ) 9 years
( ) 10 years
( ) 11 years
( ) 12 years
( ) 13 years
( ) 14 years

19) What is the sex of your cat?
( ) Male neutered
( ) Male not-neutered
( ) Female neutered
( ) Female not-neutered

20) Caring for my cat is:*
( ) Much more difficult than before the confinement ( ) More difficult than before the confinement ( ) Neither harder nor easier than before the confinement ( ) Easier than before the confinement ( ) Much easier than before the confinement

21) My cat gives me a reason to get up in the morning.*
( ) Much more than before the confinement ( ) More than before the confinement ( ) No more or less than before the confinement ( ) Less than before the confinement ( ) Much less than before the confinement

22) How often do you kiss your cat?*
( ) Much more than before the confinement ( ) More than before the confinement ( ) No more or less than before the confinement ( ) Less than before the confinement ( ) Much less than before the confinement

23) There are major aspects of owning a cat I don't like.*
( ) Much more than before the confinement ( ) More than before the confinement ( ) No more or less than before the confinement ( ) Less than before the confinement ( ) Much less than before the confinement

24) I wish my cat and I never had to be apart.*
( ) Much more than before the confinement ( ) More than before the confinement ( ) No more or less than before the confinement ( ) Less than before the confinement ( ) Much less than before the confinement

25) I feel that my cat makes too much mess.*
( ) Much more than before the confinement ( ) More than before the confinement ( ) No more or less than before the confinement ( ) Less than before the confinement ( ) Much less than before the confinement

26) How often do you play games with your cat?*
( ) Much more than before the confinement ( ) More than before the confinement ( ) No more or less than before the confinement ( ) Less than before the confinement ( ) Much less than before the confinement

27) It bothers me that my cat stops me doing things I enjoyed before I owned it.*
( ) Much more than before the confinement ( ) More than before the confinement ( ) No more or less than before the confinement ( ) Less than before the confinement ( ) Much less than before the confinement

28) How often do you spend time enjoying watching your cat?*
( ) Much more than before the confinement ( ) More than before the confinement ( ) No more or less than before the confinement ( ) Less than before the confinement ( ) Much less than before the confinement

29) It is annoying that sometimes I have to change my plans because of my cat.*
( ) Much more than before the confinement ( ) More than before the confinement ( ) No more or less than before the confinement ( ) Less than before the confinement ( ) Much less than before the confinement

30) My cat costs too much money.*
( ) Much more than before the confinement ( ) More than before the confinement ( ) No more or less than before the confinement ( ) Less than before the confinement ( ) Much less than before the confinement

31) My cat is constantly attentive to me.*
( ) Much more than before the confinement ( ) More than before the confinement ( ) No more or less than before the confinement ( ) Less than before the confinement ( ) Much less than before the confinement

32) How often do you give your cat food treats?*

( ) Much more than before the confinement ( ) More than before the confinement ( ) No more or less than before the confinement ( ) Less than before the confinement ( ) Much less than before the confinement

33) How often do you tell your cat things you don't tell anyone else?*
( ) Much more than before the confinement ( ) More than before the confinement ( ) No more or less than before the confinement ( ) Less than before the confinement ( ) Much less than before the confinement

34) How often do you feel that looking after your cat is a chore?*
( ) Much more than before the confinement ( ) More than before the confinement ( ) No more or less than before the confinement ( ) Less than before the confinement ( ) Much less than before the confinement

35) How often do you talk to your cat?*
( ) Much more than before the confinement ( ) More than before the confinement ( ) No more or less than before the confinement ( ) Less than before the confinement ( ) Much less than before the confinement

36) How often does your cat stop you doing things you want to?*
( ) Much more than before the confinement ( ) More than before the confinement ( ) No more or less than before the confinement ( ) Less than before the confinement ( ) Much less than before the confinement

37) I would like to have my cat near me all the time.*
( ) Much more than before the confinement ( ) More than before the confinement ( ) No more or less than before the confinement ( ) Less than before the confinement ( ) Much less than before the confinement

38) How often do you groom your cat?*
( ) Much more than before the confinement ( ) More than before the confinement ( ) No more or less than before the confinement ( ) Less than before the confinement ( ) Much less than before the confinement

39) If everyone else left me, my cat would still be there for me.*
( ) Much more than before the confinement ( ) More than before the confinement ( ) No more or less than before the confinement ( ) Less than before the confinement ( ) Much less than before the confinement

40) How often do you feel that having a cat is more trouble than it's worth?*
( ) Much more than before the confinement ( ) More than before the confinement ( ) No more or less than before the confinement ( ) Less than before the confinement ( ) Much less than before the confinement

41) My cat helps me get through tough times.*
( ) Much more than before the confinement ( ) More than before the confinement ( ) No more or less than before the confinement ( ) Less than before the confinement ( ) Much less than before the confinement

42) How often do you cuddle your cat?*

( ) Much more than before the confinement ( ) More than before the confinement ( ) No more or less than before the confinement ( ) Less than before the confinement ( ) Much less than before the confinement

43) My cat provides me with constant companionship.*
( ) Much more than before the confinement ( ) More than before the confinement ( ) No more or less than before the confinement ( ) Less than before the confinement ( ) Much less than before the confinement

44) How often do you have your cat with you while relaxing, i.e. watching TV?*
( ) Much more than before the confinement ( ) More than before the confinement ( ) No more or less than before the confinement ( ) Less than before the confinement ( ) Much less than before the confinement

45) My cat is there whenever I need to be comforted.*
( ) Much more than before the confinement ( ) More than before the confinement ( ) No more or less than before the confinement ( ) Less than before the confinement ( ) Much less than before the confinement

46) How often do you pet your cat?*
( ) Much more than before the confinement ( ) More than before the confinement ( ) No more or less than before the confinement ( ) Less than before the confinement ( ) Much less than before the confinement

47) How often do you buy your cat presents?*
( ) Much more than before the confinement ( ) More than before the confinement ( ) No more or less than before the confinement ( ) Less than before the confinement ( ) Much less than before the confinement

48) To what extent do you think the confinement period has affected your cat's quality of life?*
( ) It's much better
( ) It's moderately better
( ) It's slightly better
( ) It's no different
( ) It's slightly worse
( ) It's moderately worse
( ) It's much worse

49) How has the confinement period affected your relationship with your cat?*
( ) It's much better
( ) It's moderately better
( ) It's slightly better
( ) It's no different
( ) It's slightly worse
( ) It's moderately worse
( ) It's much worse

50) Do you feel that your cat helps you to better go through the period of isolation?
( ) Very much
( ) A lot
( ) Enough
( ) Neither yes nor no

( ) A little bit
( ) Little
( ) Very Little

51) Recently, how often have you been mad at your cat?*
( ) Much more than before isolation
( ) Moderately more than before isolation
( ) Slightly more than before the confinement
( ) No more and no less than before the isolation
( ) Slightly less than before the isolation
( ) Moderately less than before the confinement
( ) Much less than before the confinement

52) The following problematic behaviours are relatively common in cats. Please indicate how you have observed them in your cat.*

	**My cat never did this**	**The same as before the confinement**	**It improved during the confinement**	**It got worse during the confinement**
Aggression towards family members (hissing, scratching etc.)	( )	( )	( )	( )
Aggressiveness towards other cats living in the house	( )	( )	( )	( )
Aggression towards people who do not live in the house	( )	( )	( )	( )
Aggression towards other cats outside the house	( )	( )	( )	( )
Urination/defecation inside the house outside the litterbox	( )	( )	( )	( )
Fear of loud or unexpected noises	( )	( )	( )	( )
Destructiveness (e.g. scratching furniture)	( )	( )	( )	( )
Hiding and avoiding in contact with people	( )	( )	( )	( )

Urine marking	( )	( )	( )	( )

53) Have you observed other changes in the behaviour of your cat besides those included in the previous list?

____
____
____
____

54) What general changes have you seen in your cat's behaviour since the beginning of confinement? (please select all the options that apply)*

( ) I have not detected significant changes
( ) More nervous
( ) More stressed
( ) More relaxed
( ) More excitable
( ) More calm
( ) More attention-seeking
( ) More demanding
( ) More frustrated
( ) More irritable

Table S1 Participants' demographics and information about the household

Age groups	%	Number	Median	Min-Max
18-25	4.6	25		
26-35	23.2	127		
36-45	30.7	168		
46-55	25.2	138		
56-65	13.5	74		
66-75	2.7	15		
>76	0.2	1		
**Sex**				
Female	86.1	472		
Male	13.9	76		
**Education level**				
Middleschool diploma	3.6	20		
High school diploma	35.6	195		
Degree	39.4	216		
Post-graduate degree	20.6	113		
Other	0.7	4		
**Employment**				
Employee	27.7	152		
Freelance	17.9	98		

Student	4.9	7		
Labourer	4.2	23		
Retired	4.4	24		
Stay-at-home	4.6	25		
I work with animals (veterinarian, dog trainer etc)	18.4	101		
Other	17.9	98		
**Role in the family**				
Son/daughter	13	71		
Mother/father	33.6	184		
Adult (without children)	48.7	267		
Other	4.7	26		
**Number of people that live in the house**				
Children (0-5)			0	0-6
Children (6-12)			0	0-4
Children (13-17)			0	0-4
Adults (18-64)			2	0-5
Elderly (>65)			0	0-4
**Number of animals that live in the house**				
Dog			0	0-6
Cat			2	1-6
**Type of house**				
Flat	61.5	337		
Independent house	38.5	211		
**Size of the house**				
Small	29.7	163		
Medium to large	70.3	385		
**Perceived income**				
Low	4	22		
Mean	73	400		
High	23	126		
**Is the house big enough?**				
Yes	86.1	472		
No	13.9	76		

Is there enough air in the house?				
Yes	95.1	521		
No	4.9	27		
**Family situation during lockdown**				
We are all confined and none of us work from home	18.1	99		
We are all confined and some of us work from home	19	104		
We are all confined and we all work from home	13.5	74		
Some of us are confined and some are allowed to go out for business reasons	35.8	196		
We all have permission to go out for business reasons	13.7	75		

Table S2: Participants' demographics and information about the household

Age groups	%	Number	Median	Min-Max
18-25	4.6	25		
26-35	23.2	127		
36-45	30.7	168		
46-55	25.2	138		
56-65	13.5	74		
66-75	2.7	15		
>76	0.2	1		
**Sex**				
Female	86.1	472		
Male	13.9	76		
**Education level**				

Middleschool diploma	3.6	20		
High school diploma	35.6	195		
Degree	39.4	216		
Post-graduate degree	20.6	113		
Other	0.7	4		
**Employment**				
Employee	27.7	152		
Freelance	17.9	98		
Student	4.9	7		
Labourer	4.2	23		
Retired	4.4	24		
Stay-at-home	4.6	25		
I work with animals (veterinarian, dog trainer etc)	18.4	101		
Other	17.9	98		
**Role in the family**				
Son/daughter	13	71		
Mother/father	33.6	184		
Adult (without children)	48.7	267		
Other	4.7	26		
**Number of people that live in the house**				
Children (0-5)			0	0-6
Children (6-12)			0	0-4
Children (13-17)			0	0-4
Adults (18-64)			2	0-5
Elderly (>65)			0	0-4
**Number of animals that live in the house**				
Dog			0	0-6
Cat			2	1-6
**Type of house**				
Flat	61.5	337		
Independent house	38.5	211		
**Size of the house**				
Small	29.7	163		
Medium to large	70.3	385		

**Perceived income**		
Low	4	22
Mean	73	400
High	23	126
**Is the house big enough?**		
Yes	86.1	472
No	13.9	76
**Is there enough air in the house?**		
Yes	95.1	521
No	4.9	27
**Family situation during lockdown**		
We are all confined and none of us work from home	18.1	99
We are all confined and some of us work from home	19	104
We are all confined and we all work from home	13.5	74
Some of us are confined and some are allowed to go out for business reasons	35.8	196
We all have permission to go out for business reasons	13.7	75

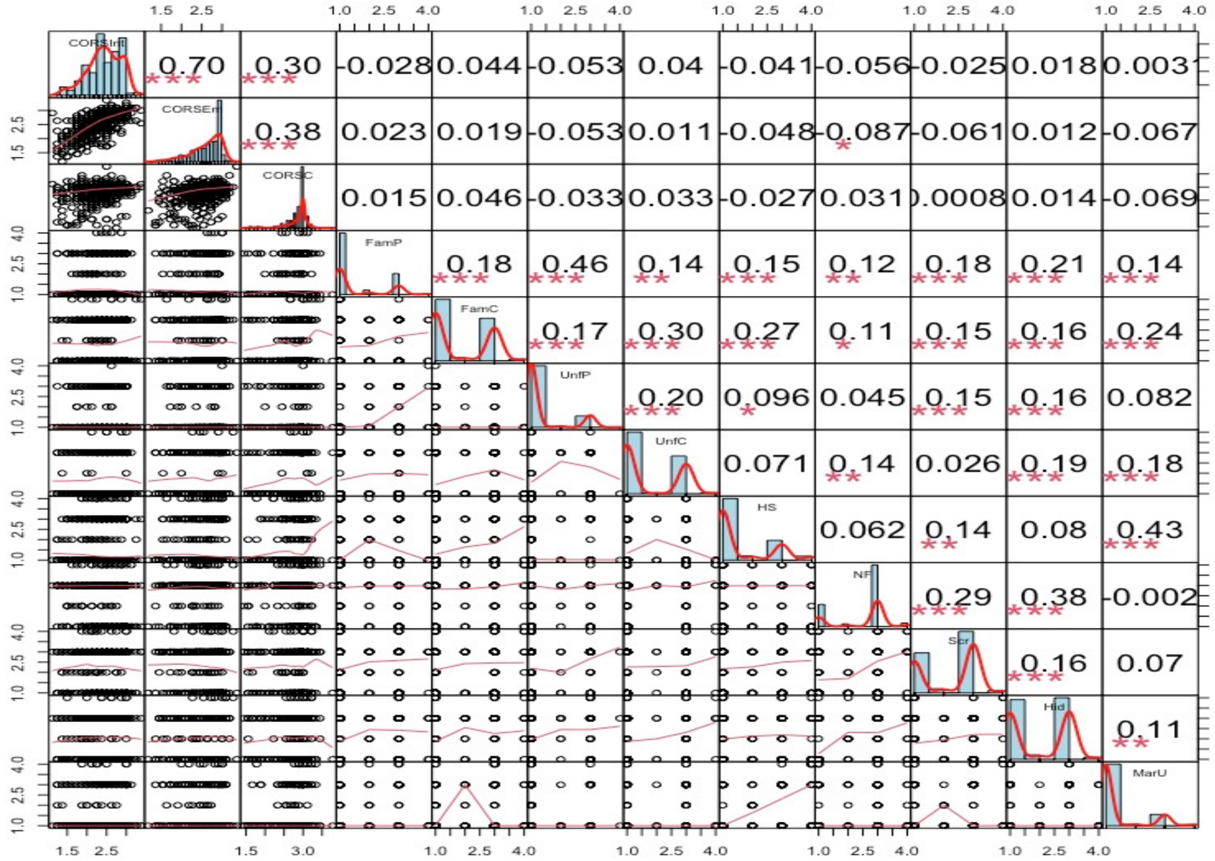

Figure S1: Scatterplot graph of the Spearman's correlations. The distribution of each variable is shown on the diagonal. On the bottom of the diagonal are reported the bivariate scatter plots with a fitted line are displayed. CORSInt = cat-owner interaction domain, CORSEm = emotional bond domain, CORSCost = perceived costs domain, FamP = aggression towards familiar people, FamC = aggression towards familiar cats, UnfP = aggression towards unfamiliar people, UnfC = aggression towards unfamiliar cats, HS = house soiling, NF = noise fear, Scr = scratching, Hid = hiding, MarU = marking with urine. On the top of the diagonal are reported the value of the correlation plus the significance level as asterisks. Each significance level is associated to a symbol: p-values 0 = "***", 0.001 = "**", 0.01 = "*", 0.05 = ".".
